# Women’s decision-making capacity and its association with comprehensive knowledge of HIV/AIDS in 23 sub-Saharan African countries

**DOI:** 10.1186/s13690-022-00849-8

**Published:** 2022-04-06

**Authors:** Betregiorgis Zegeye, Felix Emeka Anyiam, Bright Opoku Ahinkorah, Edward Kwabena Ameyaw, Eugene Budu, Abdul-Aziz Seidu, Sanni Yaya

**Affiliations:** 1 HaSET Maternal and Child Health Research Program, , Shewarobit Field Office, Shewarobit, Ethiopia; 2 Centre for Health and Development, University of Port Harcourt, Port Harcourt, Nigeria; 3 School of Public Health, Faculty of Health, University of Technology Sydney, Ultimo, Australia; 4 Department of Population and Health, University of Cape Coast, Cape Coast, Ghana; 5 Department of Estate Management, Takoradi Technical University, P.O.Box 256, Takoradi, Ghana; 6 Centre for Gender and Advocacy, Takoradi Technical University, P.O.Box 256, Takoradi, Ghana; 7 College of Public Health, Medical and Veterinary Sciences, James Cook University, Townsville, QLD, QLD4811 Australia; 8 School of International Development and Global Studies, Faculty of Social Sciences, University of Ottawa, 120 University Private, Ottawa, ON K1N 6N5 Canada; 9 The George Institute for Global Health, Imperial College London, London, UK

**Keywords:** Women’s decision-making, Knowledge of HIV, Sub-Saharan Africa, DHS, Global health

## Abstract

**Background:**

Globally, Human Immunodeficiency Virus (HIV) that causes Acquired Immunodeficiency Syndrome (AIDS) remains a public health problem with a higher burden in sub-Saharan African countries. Though the coverage is low in sub-Saharan Africa (SSA), comprehensive knowledge about HIV is vital for preventing and controlling the transmission of the virus. Women’s decision-making power is significantly linked with the knowledge and use of healthcare services. However, there is no available evidence on women’s decision-making capacity and comprehensive knowledge of HIV. Therefore, this study investigated the association between women’s decision-making capacity within households and comprehensive knowledge of HIV/AIDS in SSA.

**Methods:**

We derived data from the 2010 to 2020 Demographic and Health Surveys of 23 sub-Saharan African countries for the analysis. Using Stata version 14, both bivariate logistic regression and multivariate multilevel logistic regression analyses were used to examine the association between women’s decision-making capacity and comprehensive knowledge of HIV/AIDS at p ≤ 0.05. Results were reported using adjusted odds ratios (aOR) with their respective 95% confidence interval (CI).

**Results:**

The pooled results show that comprehensive HIV/AIDS knowledge among married women in the studied countries was 35.5%, from 18.3% in Chad to 77.1% in Rwanda. Regarding sub-regional distribution, comprehensive knowledge of HIV/AIDS in Southern Africa was 73.2%, followed by East Africa (52.4%), West Africa (43.6%), and Central Africa (35.5%). The study showed higher odds of comprehensive knowledge of HIV/AIDS among married women who had decision making power (yes-aOR = 1.20, 95% CI; 1.16–1.25) compared to those who had no decision-making power. Age, women and partner’s level of education, place of residence, exposure to media, HIV testing status, community socio-economic status, parity, religion, and distance to health facilities also had significant association with comprehensive HIV/AIDS knowledge.

**Conclusions:**

Comprehensive knowledge of HIV/AIDS in SSA is low but varies per country. Married women with decision-making capacity were more likely to have comprehensive knowledge of HIV compared to those with no decision-making capacity. Therefore, comprehensive knowledge of HIV/AIDS can be increased through enhancing women’s decision-making capacities, strengthening employment opportunities, socioeconomic capacities and creating awareness through media. Moreover, improving access to health care facilities working closely with religious leaders, can be considered to increase coverage of comprehensive knowledge of HIV among married women in SSA.

## Background

Globally, Human Immunodeficiency Virus (HIV) causing Acquired Immune Deficiency Syndrome (AIDS) continues as a serious public health problem, with a disproportionate burden in sub-Saharan African countries [1–5]. Since 2000, there has been a notable reduction in new infections due to mother-to-child transmission through resource allocation, intensive global determination, and strong political commitment and leadership at the country level [6]. Worldwide, since the beginning of the epidemic (1981), approximately 76 million people have been infected [1], about 37.9 million people are living with HIV/AIDS (PLWHA), and still about 1.7 million new infections are reported per year [1–4]. HIV is a leading cause of death worldwide and a leading cause of death globally among women of reproductive age [1, 5]. Around 5,000 new HIV infections per day are reported [4], and approximately 61% of these new infections occur in sub-Saharan Africa (SSA) [4]. AIDS-related illnesses are a leading cause of death among 15–49-year-old females globally (hundred thousand) [7, 8]. Globally, there are almost 870 000 new HIV infections among women and girls every year [8], and girls and women account for more than half of the PLWHA [8].

Ending AIDS by 2030 requires addressing and enhancing girls’ and women’s HIV/AIDS prevention roles by putting them at the center of the response [8]. In 2016, the United Nations General Assembly stated Political Declaration on Ending AIDS. It ensures universal access to quality healthcare, affordable and comprehensive sexual and reproductive health care and HIV services, information, and life-saving commodities for women [8].

Women are more at risk of getting infected with HIV because of many factors, including biological factors, intimate partner violence, and poor health systems such as poor access to sexual and reproductive health and limited employment opportunities [8]. For example, in high HIV prevalence settings, women experiencing intimate partner violence are 50% more likely to have acquired HIV than women who have not experienced violence [8].

Comprehensive knowledge about HIV/AIDS is essential to dramatically reduce the transmission of HIV virus since it allows people to have and implement correct information about sexual transmission of HIV and prevention methods [9]. It enables people to be aware of their HIV status and facilitate treatment, care, and support for PLWHA [9]. Despite efforts, the coverage of comprehensive knowledge of HIV/AIDS in SSA is still low [9–11]. For example, studies in Uganda [10] and Ghana [11] show that coverage of comprehensive knowledge of HIV was 51.9% and 59% respectively.

Several evidence show that socio-demographic, economic, and attitude-related factors influence comprehensive knowledge of HIV/AIDS [9–14]. Women’s decision-making capacity has shown to improve knowledge and access to health services [15, 16], including HIV testing [17–19]. However, there is a lack of evidence in SSA about the influence of women’s decision-making capacity and comprehensive knowledge about HIV/AIDS. Therefore, the present study examined the association between women’s decision-making capacity and comprehensive knowledge of HIV/AIDS in SSA.

## Methods

### Data source

We used data from nationally representative Demographic and Health Surveys (DHSs) of 23 sub-Saharan African countries. The study used DHS datasets spanning 2010 to 2020, and all married women aged between 15–49 years were considered. The DHS data are usually collected every five years using pretested validated quantitative data collection tools and structured methodologies. The multi-country analysis is possible due to the consistency of the method of the survey over time and across countries. Including comprehensive knowledge about HIV/AIDS, data are collected on a wide range of public health-related issues such as demographic, socioeconomic, anthropometric factors, maternity history, family planning, and domestic violence [20]. The survey involves under-5 children, men, and women aged between 15–49 years residing in non-institutional settings. Financial and technical supports for the surveys are usually provided by the United States Agency for International Development (USAID) and Inner-City Fund (ICF) International [21].

A stratified two-stage cluster sampling design is used to draw household samples. In the first stage, clusters are selected using a probability proportional to size (PPS) sampling technique. Then, in the second stage, a fixed number of households (usually 28–30 households) are selected using a systematic sampling technique [22]. The analysis was done on 182,696 married women from the individual recode (IR) file. DHS data are available in the public domain and can be accessed at http://dhsprogram.com/data/available-datasets.cfm. A more detailed information about DHS data is published elsewhere [23]. We also followed the guidelines for Strengthening Observational studies in Epidemiology (STROBE) [24]. Table 1 below provides detailed information about selected countries, year of survey, and samples.

**Table 1:** List of studied countries, year of survey and weighted sample (*N* = 182,696)

Country	Year of survey	Sampled population	
		**Weighted Number**	**Weighted %**
1. Angola	2015/16	6,129	3.4
2. Burkina Faso	2010	13,250	7.3
3. Benin	2017/18	4,957	2.7
4. Burundi	2016/17	9,281	5.1
5. Congo Democratic Republic	2013/14	11,271	6.2
6. Cote d'Ivoire	2011/12	5,877	3.2
7. Cameroon	2018/19	7,845	4.3
8. Ethiopia	2016	8,893	4.9
9. Gabon	2012	4,650	2.5
10. Gambia	2019/20	7,908	4.3
11. Kenya	2014	18,824	10.3
12. Comoros	2012	3,173	1.7
13. Liberia	2019/20	4,432	2.4
14. Mali	2018	6,693	3.7
15. Malawi	2015/16	15,672	8.6
16. Namibia	2013	3,741	2.0
17. Rwanda	2014/15	6,862	3.8
18. Sierra-Leone	2019	9,031	4.9
19. Chad	2014/15	3,315	1.8
20. Togo	2013/14	6,127	3.3
21. Uganda	2016	11,358	6.2
22. Zambia	2018/19	7,437	4.1
23. Zimbabwe	2015	5,970	3.3
Total		**182,696**	**100.00**

### Study variables

#### Outcome variable

The outcome variable of the study was comprehensive knowledge about HIV/AIDS. Participants are considered as having comprehensive knowledge of HIV if they: Know the two primary prevention methods (use of condoms and having just one uninfected faithful partner) that reduce the chance of getting HIV; and know that a healthy-looking person can have HIV; and reject the two most common local misconceptions about HIV/AIDS transmission or prevention. That means those who responded “yes” for the three questions (Can always use condoms during sex reduce risk of getting HIV? Can have 1 sex partner only, who has no other partners reduce risk of getting HIV? Can a healthy-looking person have HIV?), and those who were responded “no” for at least two of the three questions; Can get HIV from mosquito bites? Can get HIV by sharing food with a person who has AIDS? Can get HIV by witchcraft or supernatural means? [10].

#### Explanatory variables

The key explanatory variable was women’s decision-making power. In the DHS, to indirectly examine women empowerment, women are asked three decision-making questions. These are “who decides about your own health?” “who decides to purchase large household expenses?” and “who decides when you want to visit family or relatives?” Women who usually decided either alone or together with their husbands, on all three above-mentioned decision-making parameters were considered as empowered and were given the code 1, while those who indicated otherwise were considered as not empowered and were given the code 0 [25, 26].

Other individual and community level variables were selected as control variables based on a broad literature review [10, 11, 25, 26] and their availability in the DHS datasets. The following variables were considered and included in the analysis. Individual-level control variables included: women’s age in years [15–49], women’s level of education (no formal education, primary, secondary, higher), husband’s educational level **(**no formal education, primary school, secondary school, higher), women’s occupation (not employed, professional/technical/managerial, agricultural, manual, others), religion (Muslim, Christian, other religion, no religion), ever born children (0, 1–2, 3–4, 5 +), ever tested for HIV (no, yes), sex of household head (male, female), family size (< 5, 5 +), economic status (poorest, poorer, middle, richer, richest) and media exposure (no, yes). As DHS now does not collect information on income, family wealth index was used as a proxy for economic status. It is measured mainly based on component rankings generated through principal component analysis on ownership of family assets, for example, supply of drinking water, kind of toilet facility, sort of cooking fuel, and possession of television and fridge. Based on individual rankings, households have been categorized into five classes on the wealth index: poorest, poorer, middle, richer, and richest [26, 27]. Exposure to media [newspaper, radio, or television (TV)] was assessed in terms of frequency (no exposure, less than once a week, at least once a week, and almost every day). We coded “yes” if the respondent read a newspaper or listened to the radio or watched TV for at least less than once every week, and “no” as otherwise.

Regarding community-level covariates, community level factors included and coded were as follows: distance to health facility (serious problem, not a serious problem), place of residence (urban, rural), community literacy level (low, medium, high), and community socioeconomic status (low, medium, high). Big problem was considered if a participant reported that the distance to the health centre or hospital was a big problem for her. Community socioeconomic status was computed from occupation, wealth and education of participants who resided in a given community. Principal component analysis was applied to calculate women who were unemployed, uneducated, and poor. A standardized rating was derived with an average rating of 0 and standard deviation of 1. The rankings were then segregated into tertile 1 (least disadvantaged), tertile 2 and tertile 3 (most disadvantaged), where the least rating (tertile 1) denoted greater socioeconomic status with the highest score (tertile 3) denoting lower socioeconomic status.

For community literacy, respondents who had higher than secondary school education were assumed to be literate. All other respondents were given a sentence to read. They were considered literate if they could read all or part of the sentence. Therefore, high literacy included respondents with higher than secondary education or no school/primary/secondary education and could read a whole sentence. Medium literacy was when respondents had no school/primary/secondary education and could read part of the sentence. Low literacy referred to respondents who had no school/primary/secondary education and could not read at all. These were categorized into appropriate tertiles where tertile 1 (lowest score, least disadvantaged) was high community literacy, tertile 2 (medium score) was medium community literacy, and tertile 3 (highest score, most disadvantaged) was low community literacy.

#### Statistical analyses

Using Stata version 14 software, the analysis in this study was carried out using the following steps. First, descriptive analyses, including frequencies of the explanatory and control variables, overall prevalence, and distribution of the dependent variable (comprehensive knowledge of HIV/AIDS) across explanatory variables and studied countries, were presented. Then, bivariate logistic regression was done between the explanatory and control variables and the dependent variables, to measure the crude effects of these variables on the outcome, and to select candidate control variables (set at *p*-value less than 0.05), for the multivariable multilevel logistic regression model. A multicollinearity test was done using the variance inflation factor (VIF) among all selected candidate covariates to check whether there was collinearity among them. We confirmed that there was no evidence of collinearity among the independent/control variables (Mean VIF = 2.34, Max VIF = 6.67, Min VIF = 1.04). Evidence shows variance inflation factor (VIF) values less than 10 are tolerable [28, 29]. Lastly, four different models were constructed using the multilevel logistic regression (MLLR) method to assess whether the individual and community level factors had significant associations with the outcome variable (Comprehensive knowledge about HIV/AIDS). The first model was a null model, which had no explanatory variable and covariates, and it showed variance in a comprehensive knowledge about HIV/AIDS attributed to PSU. The second model, called model I, incorporated only the individual-level factors, and the third model (Model II) included community-level factors only. The last model, (Model III), comprised both the individual and community level factors.

All four MLLR models included fixed and random effects [30, 31]. The fixed effects indicated the association between the explanatory variable and/or covariates and the outcome variable and the random effects signified the measure of variation in the outcome variable based on PSU, which is measured by Intra-Cluster Correlation (ICC) [32]. Finally, the model fitness or how the different models were fitted with the data was examined using the Akaike’s Information Criterion (AIC) [33]. We used “mlogit” command to run the MLLR models. Weighting was done to take into account the complex nature of DHS data whiles the “svyset” command was used for adjusting for disproportionate sampling.

#### Ethical considerations

We used publicly available secondary data for analysis of this study (available at: https://dhsprogram.com/data/available-datasets.cfm). Ethical procedures were done by the institutions that funded, commissioned, and managed the surveys, and no further ethical clearance was required. ICF international approved that all the DHS surveys follow the U.S. Department of Health and Human Services rules for respecting of human subjects’ rights. For more details related to ethical issues, readers can visit http://goo.gl/ny8T6X.

## Results

### Socio-demographic characteristics of respondents

As shown in Table 1, in this study, a total of 182,696 married women were included for analysis. Of them, 7.2% and 19.8% were within 15–19 years (adolescents) and 20–24 years (young) age groups, respectively. About 19.4% of the participants and 16.4% of their husbands had no formal education. Nearly 27.0% and 25.3% of the participants were not employed and lived in rural areas, respectively. More than half (50.4%) of the participants reported that distance to health facilities was a big problem, and 19.1% of participants were not exposed to media (i.e., reading a newspaper, listening to the radio, or watching television, for at least less than a week).

Regarding women’s decision-making capacity, about 65.9% of married women decided (either alone or together with their husbands) on all three decision-making parameters: their own health (75.5%), purchase large household expenses (82.2%), and visit family or relatives (89.2%).

**Fig. 1:**
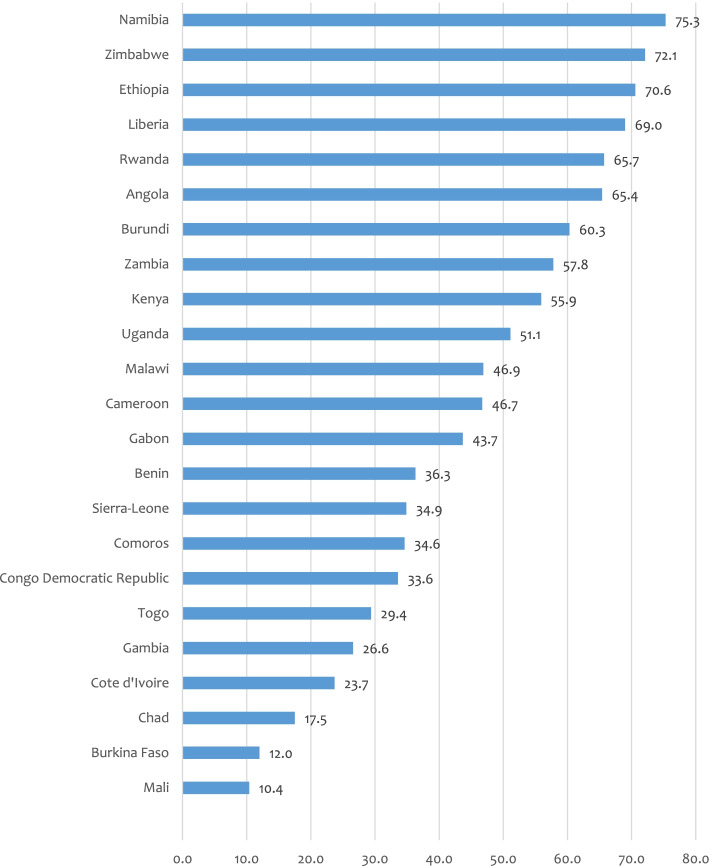
Coverage of women’s household decision making power: Evidence from 23 sub-Saharan African countries Demographic and Health Surveys (2010–2020) (*N* = 182,696)

Figure 1 shows the distribution of women’s decision-making power across studied countries. As shown in Fig. 1, women’s decision-making power varied in the studied countries, from 75.3 in Namibia, 72.1% in Zimbabwe and 70.6% in Ethiopia to 10.4% in Mali, 12% in Burkina Faso and 17.5% in Chad.

As shown in Table 2, the highest coverage of women’s decision-making capacity was observed in Southern Africa (75.3%), followed by Central Africa (65.4%), East Africa (46.7%), and West Africa (12.0%) (Table 2).

**Table 2:** Sub-regional distribution of women’s decision capacity: Evidence from 23 sub-Saharan African countries Demographic and Health Surveys countries (2010–2020) (*N* = 182,696)

Sub-regions	Included country/s	Pooled sub-regional coverage of women’s decision capacity
West Africa	Burkina Faso	12.0% (95% CI; 10.7%-13.3%)
	Benin	
	Cote d'Ivoire	
	Gambia	
	Liberia	
	Mali	
	Sierra-Leone	
	Togo	
Central Africa	Angola	65.4% (95% CI; 63.5%-67.3%)
	Congo Democratic Republic	
	Cameroon	
	Gabon	
	Chad	
East Africa	Burundi	46.7% (95% CI; 43.9%-49.5%)
	Ethiopia	
	Kenya	
	Comoros	
	Malawi	
	Rwanda	
	Uganda	
	Zambia	
	Zimbabwe	
Sothern Africa	Namibia	75.3% (95% CI; 73.1%-77.4%)

### Coverage of comprehensive knowledge of HIV

**Fig. 2:**
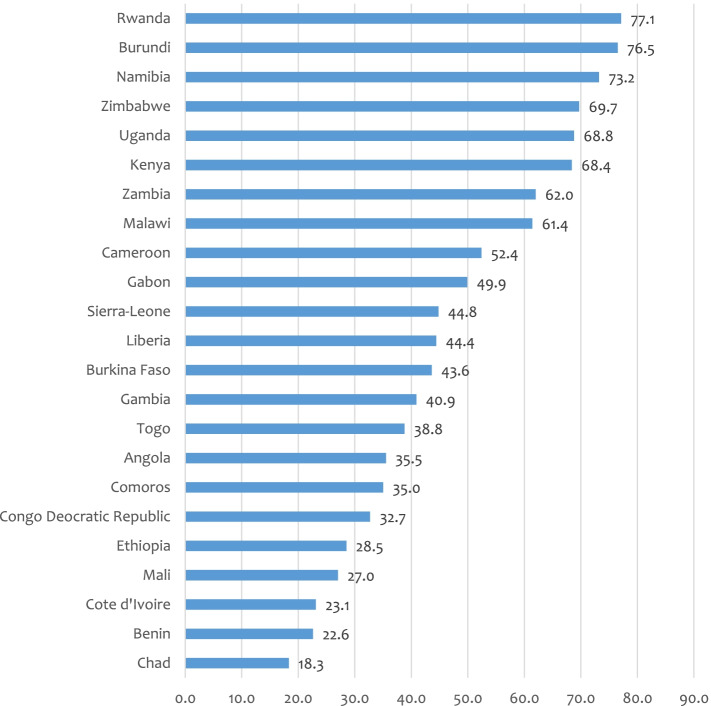
Coverage of comprehensive HIV knowledge among married women: Evidence from 23 sub-Saharan African countries DHSs (*N* = 182,696)

The pooled result shows comprehensive knowledge of HIV among married women in SSA was 35.5% with variations from 18.3%, 22.6% and 23.1% in Chad, Benin and Cote d’Ivoire respectively, to 73.2%, 76.5% and 77.1% in Namibia, Burundi and Rwanda respectively (Fig. 2).

As shown in Table 3, the highest coverage of comprehensive knowledge was seen in Southern Africa (73.2%), followed by East Africa (52.4%), West Africa (43.6%), and Central Africa (35.5%) (Table 3).

**Table 3:** Sub-regional distribution of comprehensive knowledge of HIV/AIDS among married women: Evidence from 23 sub-Saharan African countries Demographic and Health Surveys (2010–2020) (*N* = 182,696)

Sub-regions	Included country/s	Pooled sub-regional coverage of comprehensive knowledge of HIV/AIDS
West Africa	Burkina Faso	43.6% (95% CI; 41.9%-45.2%)
	Benin	
	Côte d’Ivoire	
	Gambia	
	Liberia	
	Mali	
	Sierra-Leone	
	Togo	
Central Africa	Angola	35.5% (95% CI; 32.5%-38.6%)
	Congo Democratic Republic	
	Cameroon	
	Gabon	
	Chad	
East Africa	Burundi	52.4% (95% CI; 50.1%-54.6%)
	Ethiopia	
	Kenya	
	Comoros	
	Malawi	
	Rwanda	
	Uganda	
	Zambia	
	Zimbabwe	
Sothern Africa	Namibia	73.2% (95% CI; 71.3%-75.0%)

### Comprehensive knowledge of HIV/AIDS across explanatory variables

Table 4 shows comprehensive knowledge of HIV variation across explanatory variables and subgroups. For instance, comprehensive knowledge among married women with no formal education was 18.6%, while 75.5% received a higher education. Similarly, comprehensive knowledge varied from 23.5% among married women with husbands without formal education to 63.7% among married women with husbands who had attended higher education. Comprehensive knowledge also varied from 12.6% among married women with agricultural occupation to 67.6% in those whose occupations were professional/technical/managerial, and also from 10.2% to 61.6% among married women who were from poorest and richest households, respectively (Table 4).

**Table 4:** Distribution of comprehensive knowledge of HIV across explanatory variables: Evidence from 23 sub-Saharan African countries Demographic and Health Surveys (2010–2020)

Variables	Frequency /Weighted %/	Comprehensive knowledge of HIV/AIDS (Weighted %)	COR (95% CI)
**Women’s age in years**			
15–19	11,024 (7.21)	25.49	Ref
20–24	31,823 (19.77)	33.16	1.48 (1.40–1.57)***
25–29	39,271 (22.51)	40.22	1.62 (1.53–1.71) ***
30–34	34,219 (17.41)	38.15	1.68 (1.58–1.78) ***
35–39	29,117 (14.44)	33.48	1.60 (1.50–1.70) ***
40–44	20,807 (11.65)	34.91	1.52 (1.43–1.62) ***
45–49	15,426 (7.02)	35.99	1.35 (1.26–1.45) ***
**Women’s educational level**			
No formal education	64,517 (19.44)	18.55	Ref
Primary school	67,449 (39.05)	24.81	2.15 (2.06–2.23) ***
Secondary school	42,467 (36.47)	50.49	3.32 (3.18–3.48) ***
Higher	7,244 (5.04)	75.54	7.33 (6.69–8.04) ***
**Husband’s educational level**			
No formal education	55,474 (16.40)	23.49	Ref
Primary school	51,293 (22.36)	18.54	2.06 (1.98–2.15) ***
Secondary school	50,821 (52.01)	41.59	2.21 (2.11–2.31) ***
Higher	13,064 (9.23)	63.73	3.98 (3.72–4.26) ***
**Women’s occupation**			
Not employed	45,526 (27.04)	39.59	Ref
Professional/technical/managerial	7,410 (6.57)	67.62	3.26 (3.01–3.52) ***
Agricultural	65,163 (20.87)	12.56	1.09 (1.04–1.14) ***
Manual	10,618 (3.71)	42.09	1.44 (1.35–1.54) ***
Others	42,972 (41.81)	38.71	1.14 (1.09–1.19) ***
**Economic status**			
Poorest	39,655 (11.86)	10.15	Ref
Poorer	36,579 (15.37)	12.73	1.24 (1.19–1.29) ***
Middle	35,810 (22.71)	28.79	1.50 (1.43–1.57) ***
Richer	34,415 (24.96)	41.46	1.89 (1.79–1.98) ***
Richest	35,228 (25.10)	61.60	2.97 (2.80–3.16) ***
**Media exposure**			
No	79,441 (19.07)	15.21	Ref
Yes	101,985 (80.93)	40.29	1.38 (1.33–1.43) ***
**Religion**			
Christian	116,875 (94.93)	36.21	Ref
Muslim	45,069 (0.38)	8.540	0.45 (0.42–0.47) ***
Others	4,590 (0.27)	27.87	0.41 (0.37–0.46) ***
No religion	3,634 (4.42)	23.36	0.34 (0.31–0.38) ***
**Ever born children**			
Zero	11,452 (3.70)	32.95	Ref
1–2	56,466 (31.05)	44.46	1.23 (1.17–1.30) ***
3–4	51,645 (29.30)	37.66	1.15 (1.09–1.21) ***
5 +	62,124 (35.95)	26.30	0.83 (0.78–0.87) ***
**Place of residence**			
Urban	60,999 (74.74)	43.08	Ref
Rural	120,688 (25.26)	13.12	0.66 (0.62–0.69) ***
**Household Head**			
Male	151,211 (80.96)	35.28	Ref
Female	31,485 (19.04)	36.48	1.15 (1.11–1.19) ***
**Ever tested HIV**			
No	57,111 (28.69)	14.67	Ref
Yes	124,867 (71.31)	43.90	3.52 (3.38–3.66) ***
**Family size**			
< 5	58,777 (32.60)	37.12	Ref
> = 5	122,910 (67.40)	34.73	0.80 (0.77–0.82) ***
**Distance to health facility**			
Serious problem (ref)	102,175 (50.42)	38.59	Ref
Not a serious problem	70,006 (49.58)	32.39	0.70 (0.67–0.72)***
**Decision making power**			
No	94,500 (34.07)	30.09	Ref
Yes	77,114 (65.93)	38.31	1.68 (1.62–1.74) ***
**Community literacy level**			
Low	68,013 (20.68)	12.98	Ref
Medium	61,361 (33.59)	25.71	1.36 (1.28–1.45) ***
High	53,322 (45.73)	52.90	2.12 (1.99–2.25) ***
**Community socioeconomic status**			
Low	93,138 (40.13)	16.31	Ref
Moderate	34,719 (7.74)	31.82	1.64 (1.53–1.76) ***
High	54,839 (52.13)	50.84	2.08 (1.96–2.20) ***

### Measure of association (fixed effects) results

We found a higher odds of comprehensive knowledge of HIV among married women who had decision-making power (aOR = 1.20, 95% CI; 1.16–1.25) as compared to married women who had no decision-making power. The odds of comprehensive knowledge of HIV among married women with increasing age was higher (45–49 years-aOR = 1.63, 95% CI; 1.49–1.79, 40–44 years-aOR = 1.61, 95% CI; 1.48–1.76, 35–39 years-aOR = 1.60, 95% CI; 1.47–1.75) as compared to married women aged 15–19 years. The study also shows higher odds of comprehensive knowledge of HIV among married women who had attended primary school (aOR = 1.40, 95% CI; 1.34–1.46), secondary school (aOR = 2.06, 95% CI; 1.95–2.16) and higher (aOR = 2.93, 95% CI; 2.62–3.29) as compared to married women who had no formal education. Similarly, higher odds of comprehensive knowledge was noted among married women with husbands who attended primary school (aOR = 1.18, 95% CI; 1.13–1.23) and higher (aOR = 1.11, 95% CI; 1.03–1.20) as compared to married women with husbands who had no formal education.

The study shows higher odds of comprehensive knowledge of HIV among employed married women as compared to non-employed married women. Specifically, higher odds of comprehensive knowledge was seen among married women with Professional/technical/managerial (aOR = 1.32, 95% CI; 1.21–1.44), Agricultural (aOR = 1.37, 95% CI; 1.31–1.44) and Manual (aOR = 1.22, 95% CI; 1.14–1.31) occupation as compared to unemployed married women.

Moreover, the study shows higher odds of comprehensive knowledge among married women from poorer (aOR = 1.08, 95% CI; 1.04–1.13), middle (aOR = 1.23, 95% CI; 1.17–1.29), richer (aOR = 1.35, 95% CI; 1.28–1.42) and richest (aOR = 1.58, 95% CI; 1.47–1.70) households as compared to married women from poorest households. Higher odds of comprehensive knowledge of HIV were also seen among married women who had media exposure (aOR = 1.08, 95% CI; 1.04–1.12) as compared to married women with no media exposure.

Another result from this study was lower odds of comprehensive knowledge of HIV among married women who have given birth to 3–4 children (aOR = 0.90, 95% CI; 0.83–0.96) and 5 + children (aOR = 0.75, 95% CI; 0.69–0.81) as compared to married women who had never given birth to a child. The study shows a higher odds of comprehensive knowledge of HIV among married women who have had an HIV test done (aOR = 2.51, 95% CI; 2.40–2.62) as compared to married women who had not done an HIV test. The study shows that lower odds of comprehensive knowledge of HIV among married women who belonged to the Muslim religion (aOR = 0.83, 95% CI; 0.78–0.87), other religion (aOR = 0.76, 95% CI; 0.68–0.85), and no religion (aOR = 0.61, 95% CI; 0.54–0.68) as compared to married women who were Christians.

Regarding community-level covariates, we found lower odds of comprehensive knowledge of HIV/AIDS among married women who had reported that distance to health facilities was a big problem (aOR = 0.85, 95% CI; 0.82–0.89) as compared to married women who said that distance to health facilities was not a big problem. Similarly, higher odds of comprehensive knowledge of HIV were seen among married women from rural residence (aOR = 1.27, 95% CI; 1.19–1.37) compared to married women who live in urban residence. Lastly, we found higher odds of comprehensive knowledge among married women who were from communities of medium (aOR = 1.20, 95% CI; 1.12–1.28) and high (aOR = 1.13, 95% CI; 1.04–1.23) socioeconomic status as compared to those who were from low community socioeconomic status.

### Measures of variations (random effects) results

As shown in Table 5, the results of Model 0 show that comprehensive knowledge of HIV varies significantly across the clusters (σ2 = 0.37, 95% CI; 0.32–0.44). Model 0 revealed that 8% of the prevalence of comprehensive HIV/AIDS knowledge was linked to the between-cluster variations (ICC = 0.12). The between-cluster difference decreased from 12% in Model 0 to 1% in the model that had only the Individual/household level variables (Model I). Again, the between-cluster difference increased from 1% in model I, to 4% in the community level only model (model II). Finally, ICC declined to 1% in the complete model (Model III), encompassing both individual/household and community level factors. This indicates that the differences in the probability of having comprehensive knowledge can be explained by the variances across the clusters. The values of AIC confirmed a succeeding decrease, which shows that there is a considerable improvement from empty model to individual/household and community level only models and again to the complete model. This confirms the goodness of fit of the final model established in the analysis. Hence, Model III was chosen for forecasting the association between women’s decision-making capacity and comprehensive knowledge of HIV among married women (Table 5).

**Table 5:** Multilevel multivariable logistic regression results for the association between women’s decision-making capacity and comprehensive knowledge of HIV/AIDS among married women: Evidence from 23 sub-Saharan African countries Demographic and Health Surveys (2010–2020) (*N* = 182,696)

	Model 0		Model I		Model II		Model III
**Decision making**							
No (Ref)							
Yes			1.21 (1.17–1.25)***				1.20 (1.16–1.25)***
**Women’s age in years**							
15–19 (Ref)							
20–24			1.21 (1.14–1.30)***				1.21 (1.13–1.29)***
25–29			1.36 (1.27–1.46)***				1.36 (1.27–1.46)***
30–34			1.51 (1.40–1.63)***				1.51 (1.40–1.63)***
35–39			1.60 (1.47–1.75)***				1.60 (1.47–1.75)***
40–44			1.62 (1.48–1.76)***				1.61 (1.48–1.76)***
45–49			1.63 (1.49–1.79)***				1.63 (1.49–1.79)***
**Women’s educational level**							
No formal education (Ref)							
Primary school			1.40 (1.34–1.46)***				1.40 (1.34–1.46)***
Secondary school			2.02 (1.92–2.13)***				2.06 (1.95–2.16)***
Higher education			2.88 (2.57–3.24)***				2.93 (2.62–3.29)***
**Husband’s educational level**							
No formal education (Ref)							
Primary school			1.19 (1.14–1.24)***				1.18 (1.13–1.23)***
Secondary school			1.02 (0.98–1.07)				1.04 (0.99–1.09)
Higher education			1.10 (1.02–1.19)*				1.11 (1.03–1.20)**
**Women’s occupation**							
Not employed (Ref)							
Professional/technical/managerial			1.34 (1.23–1.47)***				1.32 (1.21–1.44)***
Agricultural			1.42 (1.35–1.49)***				1.37 (1.31–1.44)***
Manual			1.24 (1.16–1.33)***				1.22 (1.14–1.31)***
Other			0.97 (0.93–1.02)				0.98 (0.94–1.03)
**Wealth index**							
Poorest (Ref)							
Poorer			1.10 (1.06–1.15)***				1.08 (1.04–1.13)***
Middle			1.26 (1.21–1.33)***				1.23 (1.17–1.29)***
Richer			1.40 (1.33–1.47)***				1.35 (1.28–1.42)***
Richest			1.64 (1.54–1.74)***				1.58 (1.47–1.70)***
**Media exposure**							
No (Ref)							
Yes			1.08 (1.04–1.12)***				1.08 (1.04–1.12)***
**Household Head**							
Male (Ref)							
Female			1.02 (0.98–1.05)				1.02 (0.98–1.06)
**Family size**							
< 5 (Ref)							
5 +			0.97 (0.94–1.01)				0.97 (0.94–1.01)
**Parity**							
No child (Ref)							
1–2			0.96 (0.90–1.02)				0.96 (0.90–1.02)
3–4			0.89 (0.83–0.96)**				0.90 (0.83–0.96)**
5 +			0.74 (0.69–0.81)***				0.75 (0.69–0.81)***
**HIV test**							
No (Ref)							
Yes			2.53 (2.43–2.64)***				2.51 (2.40–2.62)***
**Religion**							
Christian (Ref)							
Muslim			0.82 (0.78–0.86)***				0.83 (0.78–0.87)***
Others			0.75 (0.67–0.83)***				0.76 (0.68–0.85)***
No religion			0.59 (0.53–0.66)***				0.61 (0.54–0.68)***
**Distance to health facility**							
Serious problem (ref)							
Not a serious problem					0.80 (0.77–0.83)***		0.85 (0.82–0.89)***
**Place of residence**							
Urban (Ref)							
Rural					1.15 (1.07–1.23)***		1.27 (1.19–1.37)***
**Community literacy level**							
Low (Ref)							
Medium					1.16 (1.09–1.24)***		0.99 (0.93–1.05)
High					1.53 (1.41–1.65)***		1.06 (0.99–1.14)
**Community socioeconomic status**							
Low (Ref)							
Medium					1.52 (1.41–1.64)***		1.20 (1.12–1.28)***
High					1.67 (1.53–1.82)***		1.13 (1.04–1.23)**
**Random effect result**							
PSU variance (95% CI)	0.37 ( 0.32–0.44)		0.08 (0.06–0.10)		0.20 (0.16–0.26)		0.08 (0.07–0.10)
ICC	0.12		0.01		0.04		0.01
LR Test	2716.28		564.05		1163.22		590.36
Wald chi-square and *p*-value	reference		χ2 = 6784.69, *p* < 0.001		χ2 = 958.28, *p* < 0.001		χ2 = 7276.32, *p* < 0.001
**Model fitness**							
Log-likelihood	-124,703.32		-98,637.87		-115,532.46		-98,368.19
AIC	249,410.6		197,341.8		231,080.9		196,814.4
N							

*p* < 0.05; **p < 0.01; **p < 0.001

*Ref* reference category, *AIC* Akaike Information Criterion, *PSU* Primary Sampling Unit, *N* total observation, *LR* Likelihood Ratio, *ICC* Intra-class correlation coefficient

## Discussion

This study examined the association between women’s decision-making capacity and comprehensive knowledge of HIV/AIDS using data from nationally representative surveys from 23 SSA countries. Overall, the pooled results show that the coverage of comprehensive knowledge among married women in the studied countries was 35.5%, which varied from 18.3% in Chad to 77.1% in Rwanda. The overall finding is lower than a prior study in SSA (50.8%) that might be due to differences in the target population and included countries; the current study included married women and limited to 23 SSA countries while a prior study included men and 29 SSA countries [34].

We found that the likelihood of comprehensive knowledge of HIV among married women who had decision-making capacity was higher as compared to married women who had no decision-making power. Our study is consistent with a study in South Asia [35] and in Bangladesh [36]. This might be because women who had better decision-making capacity usually are more educated, have better information about HIV/AIDS [37–40], and have a less discriminatory attitude towards people with HIV [39, 41]. Several studies have documented that less decision-making capacity for women is a contributing factor in making them more susceptible to HIV [42–44]. In sub-Saharan African countries, women who have no decision-making capacity have negative attitude towards PLWHA [41, 44, 45] thereby making them more vulnerable to HIV/AIDS epidemics [9, 45]. Hence, improving women’s decision-making capacity can increase their knowledge about HIV/AIDS and change their discriminatory attitudes towards PLWHA [45].

The odds of comprehensive knowledge of HIV among married women increased with age.  This finding is in line with a prior study in Uganda [10]. The possible justification might be that older women have higher income status and better educational and carrier achievement than adolescents [46–48]. Older women are usually experienced in the utilization maternal health services, proactive and they usually emphasize on activities of prevention of diseases [46, 48, 49] and are more likely to have an HIV-test [50].

The study also shows higher odds of comprehensive knowledge of HIV/AIDS among educated married women, more specifically, among those who had attended primary school, secondary school and higher, than married women who had no formal education. The positive role of schooling for improving comprehensive knowledge was widely documented in prior studies in Iran [12], India [14], Uganda [10], Kenya [13], and eight SSA countries [9]. Education can increase knowledge about HIV/AIDS in different mechanisms [51, 52]. Education enables girls and women to have exposure to HIV/AIDS related information at school [53, 54], and it facilitates the acquisition of knowledge about maternal health services and enables women to seek healthcare services [55, 56]. This indicates that enhancing national educational coverage and ensuring that women are getting formal education could improve comprehensive knowledge of HIV for the long run [9].

Similarly, higher odds of comprehensive knowledge of HIV/AIDS was observed among married women with educated husbands as compared to married women with husbands who had no formal education. This might be partly due to the fact that husband educational level influences married women’s health care access, as documented in previous studies in Benin [57], Myanmar [58] and Bangladesh [59]. Educated husbands encourage their wives to have more access to health information and use of healthcare services [57, 60].

The present study shows higher odds of comprehensive knowledge of HIV/AIDS among employed married women as compared to not employed married women. This could be explained partly by the fact that women with job had higher self-confidence and freedom in accessing health care services that might later lead them to have awareness about health information at health facilities [59]. Most possibly, working women are more competitive both within and out of households while competing roles give women greater access to extra familiar sources of information and resources in addition to increasing their potential autonomy in family settings [59].

Moreover, the study shows higher odds of comprehensive knowledge of HIV/AIDS among married women from wealthier households as compared to married women from the poorest households. This finding is in line with prior studies in India [14], Uganda [10], Ghana [11], Ethiopia [61], Nigeria and the Republic of Congo [62] and in three east African countries (Burundi, Ethiopia, Kenya) [63]. A higher level of economy increases the likelihood of accessing information about HIV/AIDS [11]. Having better socioeconomic status improves educational achievements and media exposure, which again helps to access HIV-related information [64].

Higher odds of comprehensive knowledge of HIV were also seen among married women who had media exposure than married women with no media exposure [14, 65]. Previous studies suggest that mass media plays an influential role in promoting healthy behaviors among women [66] and increasing uptake of maternal health services [67]. The significance of mass media is influential as they may provide trustworthy and repetitive messaging to a wide-ranging audience [68] and also allow control over message content and delivery [67] at a reasonably low cost per person exposed [67]. Thus, designing media-related policies and interventions may be necessary to enhance comprehensive knowledge of HIV among married women in the region.

Another result from this study was lower odds of comprehensive knowledge of HIV/AIDS among married women who have more children as compared to married women with no child. This might be due to married women with high parity history having less exposure to health-related information because of the poor chance of accessing and utilizing health services [16, 69, 70]. That could be due to lack of time and resources caused by larger family as well as their self-confidence developed from prior pregnancy and childbirth [16, 71, 72].

The study shows higher odds of comprehensive knowledge of HIV/AIDS among married women who were tested for HIV than married women who had not tested for HIV. Consistent findings were reported in a prior study in Uganda [10] and Ethiopia [65]. The plausible reason might be due to information from healthcare providers given during counseling and testing of HIV [10]. Due to the extensive efforts of governments and donors towards accessing PMTCT services that include HIV test services, it is expected that women during pregnancy or other periods can acquire HIV-related knowledge [9].

The study shows that religion was significantly associated with comprehensive knowledge of HIV with lower odds of comprehensive knowledge of HIV among married women who belong to Muslim, other religion, and no religion compared to married women who were Christian. This might be related to the connection of HIV with and religious view [41]. The other possible justification for lower odds of comprehensive knowledge of HIV among those religious groups might be due to less access to healthcare services due to male dominance or not having permission from their husbands [73, 74]. Because of religion’s shaping and controlling capacity, human actions are usually linked with religion [75]. In African countries, religion is well thought-out as crucial to life. Hence, policy makers need to focus attention to improve maternal health services through by working diligently with religious leaders [57, 75–77].

We found lower odds of comprehensive knowledge of HIV/AIDS among married women who had reported that distance to health facilities was a serious problem as compared to married women who said that distance to health facilities was not a serious problem. The plausible reason could be long-distance, identified as one of the barriers for accessing healthcare services and related information provided by healthcare professionals [41, 57] and a significant barrier for uptake of maternal health services [78–80]. We found that, higher odds of comprehensive knowledge of HIV/AIDS among married women who were from rural residences as compared to married women who live in urban residences. This finding is inconsistent with prior studies in Uganda, Ghana, and Tanzania [81–84], and the possible reason could be due to poor access to HIV testing, poverty and lower education in rural setting. Hence, these inconsistencies need further future studies.

Lastly, we found higher odds of comprehensive knowledge among married women from communities of higher socioeconomic status than married women from low community socioeconomic status. This might be because women living in better socioeconomic environments have greater educational opportunities and media exposure as compared to women living in households or communities of lower socioeconomic [41, 61, 64]. Several scholars have reported that being unable to afford transportation costs is the main factor to not accessing health information and services [85, 86].

This study is based on a large nationally representative sample and is a multi-country study. Even so, some limitations were also observed. First, a causal-effect relationship cannot be established because of the cross-sectional nature of the study. Second, the DHS relied on self-reported data, which may be prone to recall and social desirability bias. Lastly, due to data availability and constraints, we used surveys that were conducted at different time points in the selected countries and this may affect comparison of the estimates across countries.

## Conclusion

More than one-fourth (35%) of married women had comprehensive knowledge about HIV. Married women with decision-making capacity were more likely to have comprehensive knowledge of HIV compared to those with no decision-making capacity. Therefore, enhancing women’s decision-making capacities through strengthening employment opportunities, socioeconomic capacities and creating awareness through media, accessing health facility, and working closely with religious leaders, can be considered to increase coverage of comprehensive knowledge of HIV among married women.

## Data Availability

The datasets generated and/or analyzed during the current study are available in http://dhsprogram.com/data/available-datasets.cfm.

## Abbreviations

AOR: Adjusted Odd Ratio
DHS: Demographic and Health Survey
EA: Enumeration Area
SDG: Sustainable Development Goal
WHO: World Health Organization

## References

[1] The Global HIV/AIDS Epidemic. 2021. Available at: https://www.kff.org/global-health-policy/fact-sheet/the-global-hivaids-epidemic/. Accessed 3 Mar 2021.

[2] UNAIDS. 2020 Global AIDS Update: Seizing the moment; July 2020. UNAIDS. AIDS Info website. Available at: https://aidsinfo.unaids.org/. Accessed 3 Mar 2021.

[3] UNAIDS (2020). Global HIV & AIDS statistcs-2020 fact sheet.

[4] UNAIDS data 2019. Available at: https://www.unaids.org/sites/default/files/media_asset/2019-UNAIDS-data_en.pdf. Accessed 25 Mar 2021.

[5] UNAIDS (2019). Women and HIV-A Spotlight on adolescent girls and young women.

[6] Idele P, Gillespie A, Porth T, Suzuki C, Mahy M, Kasedde S (2014). Epidemiology of HIV and AIDS among Adolescents: Current Status, Inequities, and Data Gaps. J Acquir Immune Defic Syndr.

[7] Global health estimates 2015: deaths by cause, age, sex, by country and by region, 2000–2015. Geneva: World Health Organization; 2016.

[8] UNAID. Women and girls and HIV. USAID, Joint United Nations Program, on HIV/AIDS. Geneva: UNAIDS; 2018.

[9] W Wang, S Alva, S Wang. DHS Analytical Studies 29: HIV-related Knowledge and Behaviors among people living with HIV/AIDS in high HIV prevalence countries in Sub-Saharan Africa. USAID ICF Inernational 2012. USAID ICF Int. 2012. Available at: http://measuredhs.com/pubs/pdf/AS29/AS29.pdf. Accessed 6 Nov 2021.

[10] Estifanos TM, Hui C, Tesfai AW, Teklu ME, Ghebrehiwet MA, Embaye KS, Andegiorgish AK (2021). Predictors of HIV/AIDS comprehensive knowledge and acceptance attitude towards people living with HIV/AIDS among unmarried young females in Uganda: a cross-sectional study BMC Women’s. Health.

[11] Fenny AP, Crentsil AO, Asuman D (2017). Determinants and Distribution of Comprehensive HIV/AIDS Knowledge in Ghana Global. J Health Sci.

[12] Zarei E, Khabiri R, Tajvar M, Nosratnejad S (2015). Knowledge of and attitudes toward HIV/AIDS among Iranian women. Epidemiol Health.

[13] Mwamwenda TS (2014). Education level and HIV/AIDS knowledge in Kenya. J HIV AIDS Res.

[14] Jha PK, Narayan P, Nair S, Ganju D, Sahu D, Pandey A (2015). An assessment of comprehensive knowledge of HIV/AIDS among slum and non-slum populations in Delhi, India. Open J Prev Med.

[15] Wado YD (2017). Women’s autonomy and reproductive healthcare-seeking behavior in Ethiopia, Women & Health.

[16] Yaya S, Zegeye B, Ahinkorah BO, Seidu A-Z, Ameyaw EK, Adjei NK (2021). Predictors of skilled birth attendance among married women in Cameroon: further analysis of 2018 Cameroon Demographic and Health Survey. Reprod Health.

[17] Bashemera DR, Nhembo MJ, Benedict G. The role of womens empowerment in influencing HIV testing. Calverton: ICF International; 2013.

[18] Asaolu IO, Gunn JK, Center KE, Koss MP, IwelunmorJI Ehiri JE. (2016). Predictors of HIV Testing among Youth in Sub-Saharan Africa: A Cross-Sectional Study. PLoS ONE..

[19] Yaya S, Shibre G, Idriss-Wheeler D, Uthman OA (2020). Women’s Empowerment and HIV Testing Uptake: A Meta-analysis of Demographic and Health Surveys from 33 Sub-Saharan African Countries. Int Matern Child Health AIDS.

[20] DHS Program. Methodology: Survey Type. Available at: https://dhsprogram.com/methodology/survey-Types/dHs.cfm. Accessed 4 Oct 2021.

[21] The DHS Program- Quality information to plan, monitor and improve population, health, and nutrition programs. Available at: https://dhsprogram.com/. Accessed 4 Oct 2021.

[22] DHS Program. Guide to DHS Statistics. Analyzing DHS data. Available at: https://dhsprogram.com/data/Guide-to-DHS-Statistics/Analyzing_DHS_Data.htm. Accessed 9 Oct 2021.

[23] The DHS Program - Quality information to plan, monitor and improve population, health, and nutrition programs. [Cited 10 Oct 2021]. Available from: https://dhsprogram.com/.

[24] von Elm E, Altman DG, Egger M, Pocock SJ, Gøtzsche PC, Vandenbroucke JP (2014). The strengthening the reporting of observational studies in epidemiology (STROBE) statement: guidelines for reporting observational studies. Int J Surg.

[25] Kishor S, Subaiya L. Understanding Women’s Empowerment: A Comparative Analysis of Demographic and Health Surveys (DHS) Data. DHS Comparative Reports No. 20. Calverton: Macro International Inc. 2008. https://dhsprogram.com/publications/publication-cr20-comparativereports.cfm. Accessed 12 Oct 2021.

[26] Croft Trevor N, Marshall Aileen M. J, Allen Courtney K, et al. Guide to DHS Statistics. Rockville: ICF; 2018.

[27] The DHS Program. Demographic and Health Survey (DHS). http://www.dhsprogram.com/topics/wealth-index/Index.cfm. Accessed 28 Aug 2021.

[28] Home | Academic Solutions | Academic Research Resources | Dissertation Resources | Data Entry and Management| Multicollinearity. Available at: https://www.statisticssolutions.com/multicollinearity/. Accessed 10 Oct 2021.

[29] Vittinghoff E, Glidden DV, Shiboski SC, McCulloch CE. Regression methods in biostatistics: linear, logistic, survival, and repeated measures models. Berlin, Heidelberg, New York.

[30] Austin PC, Merlo J (2017). Intermediate and advanced topics in multilevel logistic regression analysis. Stat Med.

[31] Gelman A, Hill J (2007). Data analysis using regression and multilevel hierarchical models.

[32] Perinetti G (2018). StaTips Part IV: Selection, interpretation and reporting of the intraclass correlation coefficient. South Eur J Orthod Dentofac Res.

[33] de-Graft Acquah H (2010). Comparison of Akaike information criterion (AIC) and Bayesian information criterion (BIC) in selection of an asymmetric price relationship. J Dev Agri Econ.

[34] Tetteh JK, Frimpong JB, Budu E, Mohammoed A, Ahinkorah BO, Seidu AA (2021). Comprehensive HIV/AIDS knowledge among men in sub-Saharan Africa: a multilevel modeling. J Biosoc Sci..

[35] Gagnon AJ, Merry L, Bocking J, Rosenberg E, Oxman-Martinez J (2010). South Asian migrant women and HIV/STIs: knowledge, attitudes and practices and the role of sexual power. Health Place.

[36] Khandoker A, Khan MMH, Ahsan N, Chowdhury MFE, Kabir M, Mori M (2006). Asssociation between Decision Autonomy and Knowledge of HIV/AIDS Prevention among ever married women in Bangladesh. J Med Sci.

[37] Ahmed S, Creanga AA, Gillespie DG (2010). Economic status, education and empowerment: implications for maternal health service utilization in developing countries. PLoS One.

[38] Obeidat RF (2016). How Can Women in Developing Countries Make Autonomous Health Care Decisions?. Womens Health Int..

[39] Iqbal S, Maqsood S, Zafar A, Zakar R, Zakar MZ, Fischer F (2019). Determinants of overall knowledge of and attitudes towards HIV/AIDS transmission among ever-married women in Pakistan: evidence from the Demographic and Health Survey 2012–13. BMC Public Health.

[40] Heaton TB, Huntsman TJ, Flake DF (2005). The effects of status on women’s autonomy in Bolivia, Peru, and Nicaragua. Popul Res Policy Rev.

[41] Zegeye B, Adjei NK, Ahinkorah BO, et al. Individual-, household-, and community-level factors associated with pregnant married women’s discriminatory attitude towards people living with HIV in sub-Saharan Africa: A multicountry cross-sectional study. Health Sci Rep. 2021;4:e430. 10.1002/hsr2.430 PMC8549109 34746443

[42] Chacham AS, Maia MB, Greco M, Silva AP, Greco DB (2007). Autonomy and susceptibility to HIV/AIDS among young women living in a slum in Belo Horizonte. Brazil AIDS Care.

[43] Bloom SS, Griffiths PL (2007). Female Autonomy as a contributing factor to women’s HIV/AIDS related knowledge and behaviour in three culturally constrasting States in India. J Biosoc Sci.

[44] Hindin MJ, Muntifering CJ (2011). Women’s autonomy and timing of most recent sexual intercourse in Sub-Saharan Africa: a multi-country analysis. J Sex Res.

[45] Rirash F, "The Association Between Women's Autonomy and Women's HIV/AIDS Knowledge and Attitudes in Ethiopia" Electronic Thesis and Dissertation Repository.1992, 2014.

[46] Kato T, Yorifuji T, Yamakawa M, Inoue S, Doi H, Eboshida A (2017). Association of maternal age with child health: a Japanese longitudinal study. PLoS ONE..

[47] Benzies K, Tough S, Tofflemire K, Frick C, Faber A, Newburn-Cook C (2006). Factors influencing women’s decisions about timing of motherhood. J Obstet Gynecol Neonatal Nurs.

[48] Zegeye B, Keetile M, Ahinkorah BO, Ameyaw EK, Seidu AA, Yaya S (2021). Utilization of deworming medication and its associated factors among pregnant married women in 26 sub-Saharan African countries: a multi-country analysis Tropical Medicine and Health.

[49] Nelson AM (2004). A qualitative study of older first-time mothering in the first year. J Pediatr Health Care.

[50] UNAID. Women and girls and HIV. USAID, Joint United Nations Program, on HIV/AIDS. Geneva: UNAIDS; 2018.

[51] Jukes M, Simmons S, Bundy D (2008). Education and vulnerability: the role of schools in protecting young women and girls from HIV in southern Africa. AIDS.

[52] Fadumo R. “The Association between Women’s Autonomy and Women’s HIV/AIDS Knowledge and Attitudes in Ethiopia” Electronic Thesis and Disser tation Repository 1992. 2014.

[53] Ochako R, Ulwodi D, Njagi P (2011). Trends and determinants of Comprehensive HIV and AIDS knowledge among urban young women in Kenya. AIDS Res Ther.

[54] Baker DP, Leon J, Collins JM (2011). Facts, Attitudes, and Health Reasoning About HIV and AIDS: Explaining the Education Effect on Condom Use among Adults in Sub-Saharan Africa. AIDS Behav.

[55] Shahabuddin A, Delvaux T, Abouchadi S, Sarker M, De Brouwere V (2015). Utilization of maternal health services among adolescent women in Bangladesh: a scoping review of the literature. Tropical Med Int Health.

[56] Tiruneh FN, Chuang K-Y, Chuang Y-C (2017). Women’s autonomy and maternal healthcare service utilization in Ethiopia. BMC Health Serv Res.

[57] Zegeye B, El-khatib Z, Ameyaw EK, Seidu A, Ahinkorah BO, Keetile M (2021). Breaking Barriers to Healthcare Access: A Multilevel Analysis of Individual- and Community-Level Factors Affecting Women’s Access to Healthcare Services in Benin. Int J Environ Res Public Health.

[58] Mie HNM, Hnin ZL, Khaing W. Empowerment and Barriers to Health Care Access among Currently Married Women: Secondary Data Analysis of the 2015–16 Myanmar Demographic and Health Survey. DHS Working Paper No. 146. Rockville: ICF; 2019.

[59] Mainuddin AKM, Bagum HA, Rawal LB, Islam A, Islam SMS (2015). Women empowerment and its relation with health seeking behavior in Bangladesh. J Fam Reprod Health..

[60] Levtov R, Van Der Gaag V, Greene M, Michael K, Barker G (2015). State of the World’s Fathers. a MenCare Advocacy Publication.

[61] Agegnehu CD, Geremew BM, Sisay MM, Muchie KF, Engida ZT, Gudayu TW (2020). Determinants of comprehensive knowledge of HIV/AIDS among reproductive age (15–49 years) women in Ethiopia: further analysis of 2016 Ethiopian demographic and health survey. AIDS Res Ther.

[62] Gebremedhin S, Wang Y, Tesfamariam E (2017). Predictors of HIV/AIDS knowledge and attitude among young women of Nigeria and Democratic Republic of Congo: cross-sectional study. J AIDS Clin Res.

[63] Teshome R, Youjie W, Habte E, Kasm N (2016). Comparison and association of comprehensive HIV/AIDS knowledge and attitude towards people living with HIV/AIDS among women aged 15–49 in three East African countries: Burundi, Ethiopia and Kenya. J AIDS Clin Res.

[64] Dimbuene ZT, Defo BK (2011). Fostering accurate HIV/AIDS knowledge among unmarried youths in Cameroon: do family environment and peers matter?. BMC Public Health.

[65] Kefale B, Damtie Y, Yalew M, Adane B, Arefaynie M (2020). Predictors of Comprehensive Knowledge of HIV/ AIDS Among People Aged 15–49 Years in Ethiopia: A Multilevel Analysis. HIV/AIDS - Res and Palliative Care..

[66] Ankomah A, Adebayo SB, Arogundade ED, Anyanti J, Nwokolo E, Inyang U, et al. The Effect of Mass Media Campaign on the Use of Insecticide-Treated Bed Nets among Pregnant Women in Nigeria. Malar Res Treat. 2014;1(1):694863. 10.1155/2014/694863 PMC3980778 24778895

[67] Zamawe CO, Banda M, Dube AN (2016). The impact of a community driven mass media campaign on the utilisation of maternal health care services in rural Malawi. BMC Pregnancy Childbirth.

[68] Asp G, Pettersson OK, Sandbeg J, Kabakyenga J, Agardh A (2014). Associations between mass media exposure and birth preparedness among women in southwestern Uganda: a community-based survey. Glob Health Action.

[69] Tekelab T, Yadecha B, Melka AS (2015). Antenatal care and women’s decision making power as determinants of institutional delivery in rural area of Western Ethiopia. BMC Res Notes.

[70] Fawole OI, Adeoye IA (2015). Women’s status within the household as a determi- nant of maternal health care use in Nigeria. Afr Health Sci.

[71] Krugu JK, Mevissen F, Münkel M, Ruiter R (2017). Beyond love: a qualitative analysis of factors associated with teenage pregnancy among young women with pregnancy experience in Bolgatanga. Ghana Cult Health Sex.

[72] Christofides NJ, Jewkes RK, Dunkle KL, McCarty F, Shai NJ, Nduna M (2014). Risk factors for unplanned and unwanted teenage pregnancies occurring over two years of follow-up among a cohort of young South African women. Glob Health Action.

[73] Fagbamigbe AF, Idemudia ES (2015). Barriers to antenatal care use in Nigeria: evidences from non-users and implications for maternal health programming. BMC Pregnancy Childbirth.

[74] Doctor HV, Findley SE, Ager A, Commeto G, Afenyadu GY, Adamu F (2012). Using community based research to shape the design and delivery of maternal health services in Northern Nigeria. Reprod Health Matters.

[75] Solankea BL, Oladosub OA, Akinloc A (2015). Olanisebed SO Religion as a Social Determinant of Maternal Health Care Service Utilisation in Nigeria. Afr Popul Stud..

[76] Heward-Mills NL, Atuhaire C, Spoors C, Pemunta NV, Priebe G, Cumber SN (2018). The role of faith leaders in influencing health behaviour: a qualitative exploration on the views of Black African christians in Leeds. UK Pan African Med J.

[77] Aziato L, Odai PNA, Omenyo CN (2016). Religious beliefs and practices in pregnancy and labour: an inductive qualitative study among post-partum women in Ghana. BMC Pregnancy Childbirth.

[78] Okedo-Alex IN, Akamike IC, Ezeanosike OB, Uneke CJ (2019). Determinants of antenatal care utilisation in sub-Saharan Africa: a systematic review. BMJ Open..

[79] Kawungezi PC, Akiibua D, Aleni C, Chitayi M, Niwaha A, Kazibwe A (2015). Attendance and utilization of antenatal care (ANC) services: multi-center study in upcountry areas of Uganda. Open J Prev Med.

[80] Shibre G, Zegeye B, Ahinkorah BO, Idriss-Wheeler D, Keetile M, Yaya S (2021). Sub-regional disparities in the use of antenatal care service in Mauritania: findings from nationally representative demographic and health surveys (2011–2015). BMC Public Health.

[81] Ankunda D (2017). Determinants of comprehensive knowledge of HIV/AIDS among women of the reproductive age (15–49) in Uganda.

[82] Fenny AP, Crentsil AO, Asuman D (2017). Determinants and distribution of comprehensive HIV/AIDS knowledge in Ghana. Glob J Health Sci.

[83] Epsley EJ, Nhandi B, Wringe A, Urassa M, Todd J (2011). Evaluation of knowledge levels amongst village AIDS committees after undergoing HIV educational sessions: results from a pilot study in rural Tanzania. BMC Int Health Hum Rights.

[84] Abate BB, Kassie AM, Reta MA, Ice GH, Haile ZT (2020). Residence and young women’s comprehensive HIV knowledge in Ethiopia. BMC Public Health.

[85] Dussault G, Franceschini MC (2006). Not enough there, too many here: understanding geographical imbalances in the distribution of the health workforce. Hum Resour Health.

[86] Berhane Y, Gossaye Y, Emmelin M (2001). Women’s health in a rural setting in societal transition in Ethiopia. Soc Sci Med.

